# Mixed Valence of Ce and Its Consequences on the Magnetic State of Ce_9_Ru_4_Ga_5_: Electronic Structure Studies

**DOI:** 10.3390/ma13102377

**Published:** 2020-05-21

**Authors:** Andrzej Ślebarski, Józef Deniszczyk, Dariusz Kaczorowski

**Affiliations:** 1 Institute of Physics, University of Silesia in Katowice, 75 Pułku Piechoty 1, 41-500 Chorzów, Poland; 2 Institute of Materials Engineering, University of Silesia in Katowice, 75 Pułku Piechoty 1A, 41-500 Chorzów, Poland; jozef.deniszczyk@us.edu.pl; 3 Institute of Molecular Physics, Polish Academy of Sciences, Mariana Smoluchowskiego 17, 60-179 Poznań, Poland; kaczorowski@ifmpan.poznan.pl or; 4 Institute of Low Temperature and Structure Research, Polish Academy of Sciences, PO Box 1410, 50-950 Wrocław, Poland

**Keywords:** intermetallic Ce compounds, X-ray photoelectron spectroscopy, electronic band structure, hybridization, valence instability, density functional theory

## Abstract

We report on X-ray photoelectron spectroscopy (XPS) and ab initio electronic structure investigations of a novel intermetallic material Ce${}_{9}$Ru${}_{4}$Ga${}_{5}$. The compound crystallizes with a tetragonal unit cell (space group I4mm) that contains three inequivalent Ce atoms sites. The Ce 3d core level XPS spectra indicated an intermediate valence (IV) of selected Ce ions, in line with the previously reported thermodynamic and spectroscopic data. The ab initio calculations revealed that Ce1 ions located at 2a Wyckoff positions possess stable trivalent configuration, whereas Ce2 ions that occupy 8d site are intermediate valent. Moreover, for Ce3 ions, located at different 8d position, a fractional valence was found. The results are discussed in terms of on-site and intersite hybridization effects.

## 1. Introduction

Physical properties of Ce-based intermetallic compounds are mainly determined by two competing interactions: Kondo effect, characterized by a temperature $T_{K}\propto exp\left(-\frac{1}{|J_{fc}N\left(E_{F}\right)|}\right)$, and Ruderman–Kittel–Kasuya–Yosida (RKKY) interaction, related to $T_{RKKY}\propto J_{fc}^{2}N\left(E_{F}\right)$. In both expressions, $N(E_{F})$ stands for density of states (DOS) at Fermi level $E_{F}$, and $J_{fc}∼V_{fc}$ is the coupling constant between 4f and conduction (c) electron states, where $V_{fc}$ represents on-site hybridization energy given by *f*–*c* hybridization matrix element. According to the Schrieffer–Wolff transformation [1], $J_{fc}$ is defined as $J_{fc}=\frac{2V_{fc}^{2}}{|E_{f}-E_{F}|}$, where $E_{4f}$ stands for energy of 4f level. The energy $V_{fc}$ determines filling of the 4f shell, and thus governs the character of magnetic ground state. In the Ce-based intermetallics, the hybridization $V_{fc}$ results in a variety of intriguing properties such as heavy-fermion behavior, unconventional superconductivity, various magnetic ordering, non-Fermi liquid, and quantum critical phenomena [2].

For a number of Ce-based compounds reported in the literature, Ce ions occupy a single position in their crystallographic unit cells. If the 4f-electron states are strongly localized, i.e., the Kondo interaction is weak, generally, a kind of magnetic ground state is expected. Most often, the compounds order antiferromagnetically, yet, a few ferromagnets are also known, e.g., Ce${}_{2}$RuGe${}_{2}$ [3], CeRuPO [4], Ce${}_{3}$RhSi${}_{3}$ [5], CePd${}_{2}$Al${}_{8}$ [6], CeCrGe${}_{3}$ [7], or Ce${}_{11}$Pd${}_{4}$In${}_{9}$ [8]. However, the situation becomes less obvious when there is more than a single inequivalent Ce site in the crystal structure. Different local environments of the Ce ions can lead to dissimilar hybridization strengths, which spark the possibility of having distinctly different ground states for each individual inequivalent Ce ion. Recently, investigation of Ce-based compounds bearing multiple inequivalent Ce sites has received considerable attention, and for a few of them, diverse unusual low-temperature properties were established. Prominent examples are Ce${}_{5}$Ni${}_{6}$In${}_{11}$, with separate antiferromagnetic orderings in two different Ce atom sublattices [9]; Ce${}_{3}$Pd${}_{20}$Si${}_{6}$, with dipolar and quadrupolar antiferromagnetic orders associated with inequivalent Kondo sites [10]; or Ce${}_{3}$PtIn${}_{11}$ and Ce${}_{3}$PdIn${}_{11}$, where two different Ce atom sublattices host antiferromagnetism and heavy-fermion superconductivity [11,12,13,14].

Another exciting case is the coexistence in a single material of long-range magnetic ordering and valence fluctuations, each phenomenon emerging in a separate Ce atom sublattice. Recently, this very rare situation was reported to occur, e.g., in Ce${}_{2}$RuGe [15], with two independent Ce atom sites in its crystallographic unit cell, and Ce${}_{9}$Ru${}_{4}$Ga${}_{5}$, which possesses as many as three inequivalent Ce atom sublattices [16,17]. Remarkably, in both compounds one of the different Ce atoms is coordinated by its Ru neighbors at a distance of ∼2.2 Å and ∼2.4 Å, respectively, which is much shorter than the sum of the covalent radii of the Ce and Ru atoms. As discussed in detail in a series of our previous papers on the Ce-Ru-*X* intermetallics (*X* = Ge, Ga, Al) [15,16,17,18,19,20,21,22], strong Ce–Ru bonding brings about a significant instability of the electronic 4f shell, and thus intermediate valence behavior may arise. At the same time, the Ce ions with Ce-Ru distances of regular length remain their trivalent character that promotes localized magnetism with possible magnetic ordering at low temperatures.

The present research was aimed at verification of the electronic character of the particular Ce ions in Ce${}_{9}$Ru${}_{4}$Ga${}_{5}$ by means of X-ray photoelectron spectroscopy and ab initio band structure calculations. Our results fully support the scenario of the dual nature of the 4f electrons in this material.

## 2. Experimental and Computational Details

X-ray photoelectron spectroscopy (XPS) experiments were carried out on a polycrystalline sample Ce${}_{9}$Ru${}_{4}$Ga${}_{5}$ used before for magnetization, magnetic susceptibility, specific heat, and resistivity measurements [17]. The XPS spectra were obtained at room temperature in vacuum of ~$10^{-10}$ Torr using a Physical Electronic PHI 5700/600 ESCA spectrometer (Physical Electronics, Inc., Chanhassen, MN, USA) with monochromatized Al Kα radiation. The sample was broken in high vacuum of $6\times 10^{-10}$ Torr, immediately before the spectra were recorded. Calibration of the spectral data was performed in a manner described in [23]. Binding energies were referenced to the Fermi level ($E_{F}=0$).

The electronic band structure of Ce${}_{9}$Ru${}_{4}$Ga${}_{5}$ was calculated using the full-potential linearized augmented plane waves (FP-LAPW) method [24] implemented in the WIEN2k computer code (WIEN2k_18.1, released on 30 June 2018, Institute of Materials Chemistry, TU Viena, Austria) [25] (for details on similarly made computations see, e.g., in [26]). In the performed calculations, we assumed the following electronic configurations of strongly-bound core level states (SC), weakly-bound states (WC), and valence band states in the particular atoms; Ce: [Kr]${}_{\mathrm{SC}}${$4d^{10}5s^{2}5p^{6}$}${}_{\mathrm{WC}}$($4f^{1}5d^{1}6s^{2}$)${}_{\mathrm{VB}}$; Ru: [Ar + $3d^{10}$]${}_{\mathrm{SC}}${$4s^{2}4p^{6}$}${}_{\mathrm{WC}}$($4d^{7}5s^{1}$)${}_{\mathrm{VB}}$; and Ga: [Ne+$3s^{2}$]${}_{\mathrm{SC}}${$3p^{6}3d^{10}$}${}_{\mathrm{WC}}$($4s^{2}4p^{1}$)${}_{\mathrm{VB}}$. The fully relativistic formalism was implemented for the SC states, while local orbital (LO) states and VB electrons were treated within the scalar-relativistic Kohn–Sham approach. The spin-orbit (SO) interaction was applied within the second variational approach [24] for calculation of the VB and LO states. The revised Perdew–Burke–Ernzerhof (PBEsol) generalized gradient approximation (GGA) [27] was applied for the exchange correlation (XC) potential.

**Figure 1:**
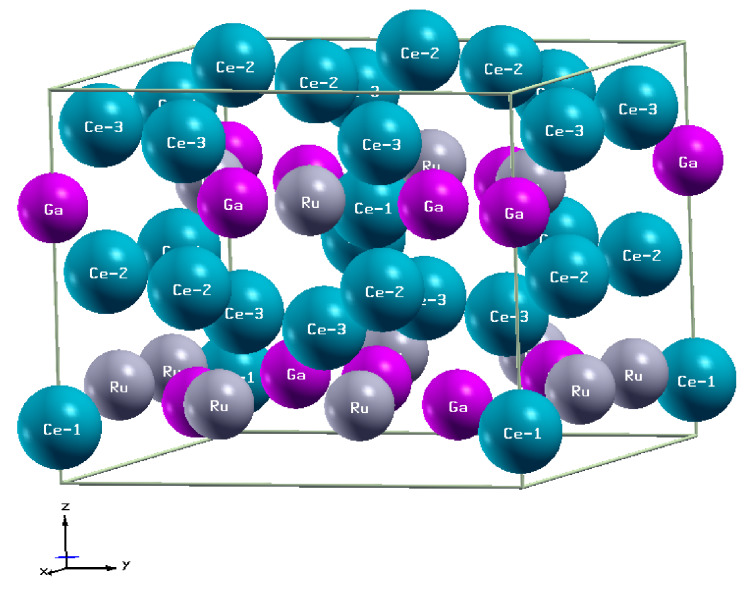
Tetragonal unit cell of Ce${}_{9}$Ru${}_{4}$Ga${}_{5}$ (space group I4*mm*, No 107). The structure details are given in Table 1.

To determine theoretically the magnetic properties of the individual Ce ions in Ce${}_{9}$Ru${}_{4}$Ga${}_{5}$, the following procedure was applied. First, the PBEsol XC potential was corrected by the Hubbard-like correlation interaction using the approach developed by Anisimov et al. [28,29] with correlation energy parameter *U* = 1.5, 2.25, and 3 eV [30]. Then, the ab initio calculations were made within FP-LAPW approach, assuming the muffin-tin (MT) model for crystal potential. The radii of MT spheres, $R_{\mathrm{MT}}$, were taken equal 0.121 nm, 0.101 nm, and 0.111 nm for Ce, Ru, and Ga ions, respectively. The accuracy of the performed calculations was determined by the following parameters; $l_{\max}=10$, $G_{\max}=14$, and $K_{\max}=9/R_{\mathrm{MT}}≃8.17\;{\mathrm{nm}}^{-1}$. A number of 324$\vec{k}$ vectors in the irreducible Brillouin zone used in the calculations was found to ensure a total energy convergence of the order of 0.01 eV. The structural data assumed in the initial calculations were taken from work in [16]; however, an atomic relaxation was performed to reach the equilibrium structure. Figure 1 shows the crystal structure of Ce${}_{9}$Ru${}_{4}$Ga${}_{5}$, which was the basis for our calculations. In the crystallographic unit cell, there are three inequivalent Wyckoff positions for cerium atoms: 2a site with Ce1 atoms, 8d site with Ce2 atoms, and another 8d site with Ce3 atoms [16]. Throughout the present paper we adopted the Ce atoms labels introduced in Table 2 in Ref. [16]. One should note, however, that in the text of the latter publication and in its figures the Ce1 atom was mistakenly switched with the Ce2 atom (we thank Dr. Elena Murashova, a coauthor of Ref. [16], for giving us comprehensive information about that error).

## 3. XPS Results

**Figure 2:**
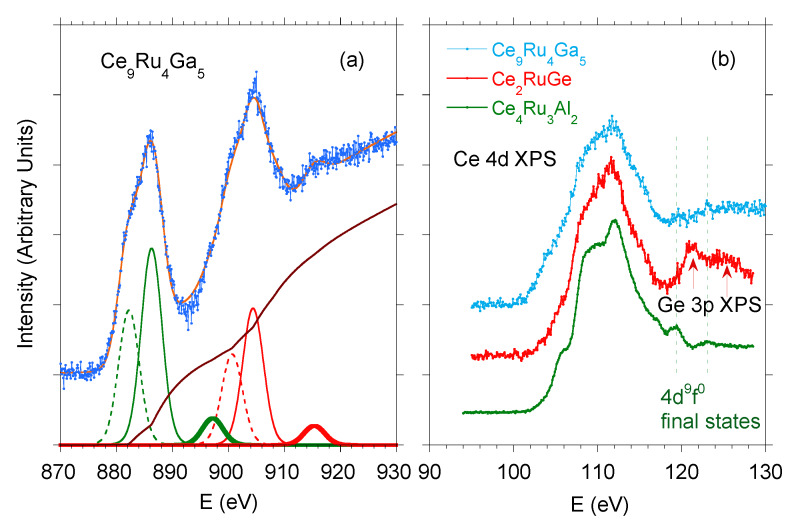
(**a**) Experimental Ce 3d core-level X-ray photoelectron spectroscopy (XPS) spectrum of Ce${}_{9}$Ru${}_{4}$Ga${}_{5}$ (blue points) and its Gunnarsson–Schönhammer (GS) modeling (orange line). The contributions $3d_{5/2}^{9}4f^{n}$ and $3d_{3/2}^{9}4f^{n}$ (with *n* = 0, 1, and 2) are presented in green and red, respectively. The estimated SO splitting is 18.6 eV. The components $3d^{9}4f^{1}$, $3d^{9}4f^{2}$, and $3d^{9}4f^{0}$ are marked by solid, dashed and thick curves, respectively. The brown line represents the calculated background. Panel (**b**) shows Ce 4d XPS spectrum of Ce${}_{9}$Ru${}_{4}$Ga${}_{5}$ compared with the respective spectra of the similar intermediate valent compounds Ce${}_{2}$RuGe and Ce${}_{4}$Ru${}_{3}$Al${}_{2}$. For each compound, two features located at 120 and 124 eV (marked by vertical dotted lines) signal mixed valence of Ce ions.

The X-ray absorption near-edge structure (XANES) spectroscopy, performed for Ce${}_{9}$Ru${}_{4}$Ga${}_{5}$ near its Ce $L_{3}$ edge, revealed a mixed valence state of the Ce ions, giving an average valence of Ce ions to be about 3.1 at room temperature [16]. In order to corroborate that finding, we measured Ce 3d and Ce 4d core-level XPS spectra and analyzed the results in terms of the Anderson theory [31]. For a system with partial filling of the Ce 4f shell, the theory predicts the appearance of the $f^{0}$ and $f^{2}$ final states as a result of intra-atomic hybridization between 4f and conduction band states. The 3d XPS spectrum recorded at room temperature is presented in Figure 2a. The main lines correspond to the $3d_{5/2}^{9}4f^{1}$ and $3d_{3/2}^{9}4f^{1}$ final states, separated by spin-orbit (SO) interaction $\Delta_{\mathrm{SO}}=18.6$ eV. Most remarkably, the spectrum also shows satellites $3d_{5/2}^{9}4f^{n}$ and $3d_{3/2}^{9}4f^{n}$ with *n* = 0 and 2, separated by the same energy $\Delta_{\mathrm{SO}}$.

According to the Gunnarsson–Schönhammer (GS) model [32,33], the $3d4f^{0}$ line arises due to the intermediate valence effect, whereas $3d4f^{2}$ reflects the on-site hybridization strength, which is expressed by the energy $\Delta_{fc}=\pi V_{fc}^{2}N\left(E_{F}\right)$ [31]. It is possible to separate of the overlapping peaks on the basis of the Doniach–Šunjić theory [34], and $\Delta_{fc}$ can be estimated from the intensity ratio $I\left(f^{2}\right)/\left[I\left(f^{1}\right)+I\left(f^{2}\right)\right]$ of the respective Ce 3d XPS lines [33]. In turn, the intensity ratio $r=I\left(f^{0}\right)/\left[I\left(f^{0}\right)+I\left(f^{1}\right)+I\left(f^{2}\right)\right]$ gives an estimate for the 4f shell mean occupation number $n_{f}$ [33]. The accuracy of determining $\Delta_{fc}$ and $n_{f}$ is usually less than 20% [35,36] (the limitations were discussed in details, e.g., in [33]). Moreover, one should note that these two quantities are interrelated.

In the case of Ce${}_{9}$Ru${}_{4}$Ga${}_{5}$, we found from the GS approach $\Delta_{fc}\approx 210$ meV. In order to determine the ground-state 4f occupation, we used the theoretical method proposed by Fuggle et al. in Ref. [33], where the *r* ratio is calculated as a function of the initial *f* occupation $c_{\left(f^{0}\right)}$ for different values of $\Delta_{fc}$. Assuming $n_{f}\approx 1-c_{\left(f^{0}\right)}$ and $c_{\left(f^{0}\right)}$ equal to wave function amplitude of the initial $f^{0}$ configuration state [33], we derived the fractional 4f electron count $n_{f}\approx 0.88$.

The fractional valence of Ce ions in Ce${}_{9}$Ru${}_{4}$Ga${}_{5}$ was further corroborated by inspection of the Ce 4d XPS spectrum (see Figure 2b), which exhibits two lines near 120 and 124 eV, characteristic of the Ce${}^{4+}$ states [33].

## 4. Calculated Electronic Structure

The atomic positions in the crystallographic unit cell of Ce${}_{9}$Ru${}_{4}$Ga${}_{5}$, obtained as a result of minimizing interatomic forces, are presented in Table 1, and the so-derived local environments of the Ce1, Ce2, and Ce3 atoms are given in Table 2. All the respective interatomic distances are very similar to those reported in the literature [16] (see also Ref. citeremark ).

**Figure 3:**
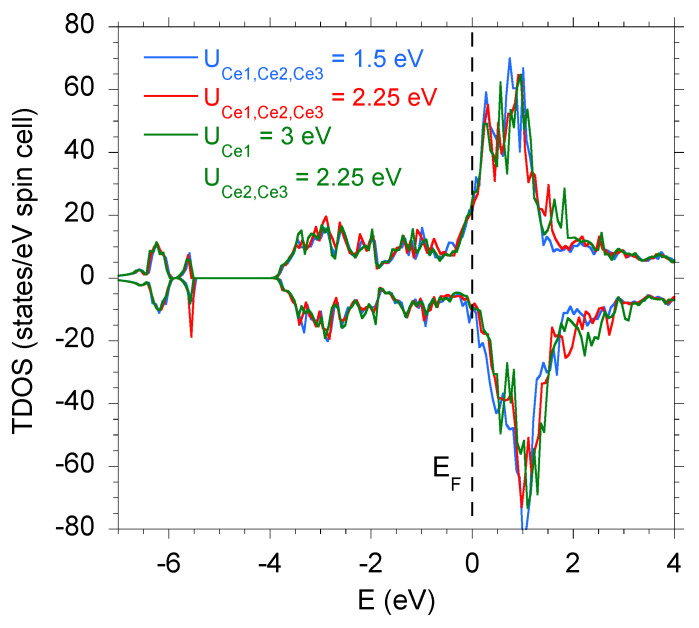
Total spin-resolved density of states in Ce${}_{9}$Ru${}_{4}$Ga${}_{5}$ calculated for different correlation energy parameter *U*.

**Figure 4:**
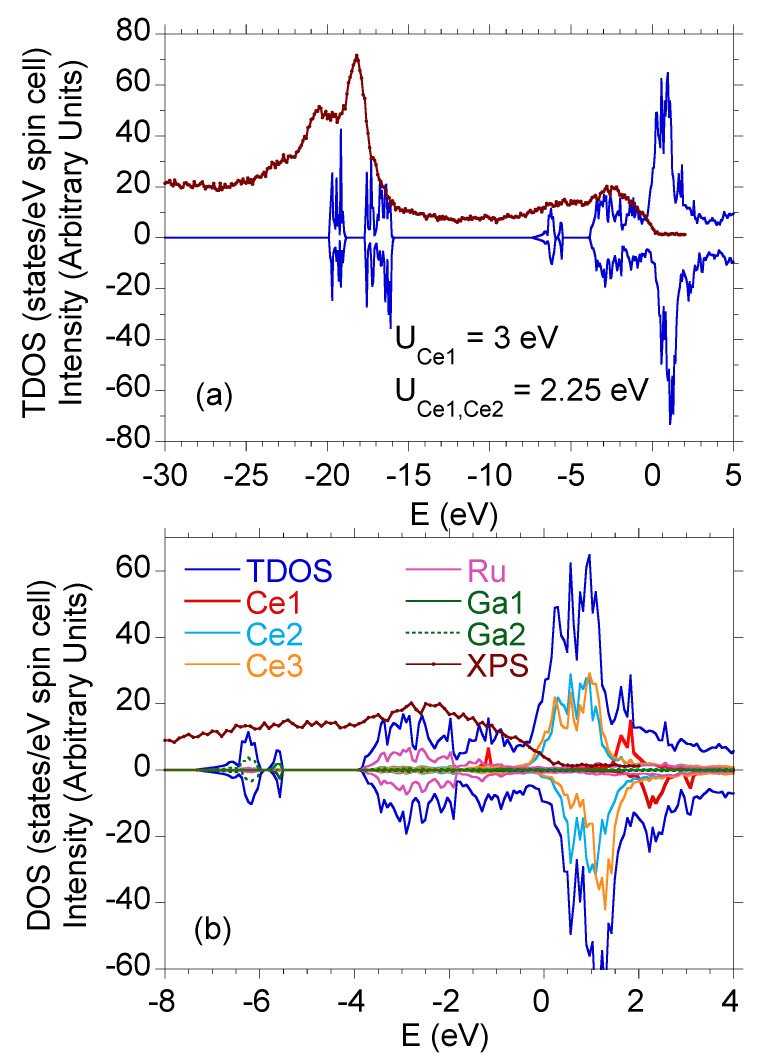
(**a**) Valence band XPS spectrum of Ce${}_{9}$Ru${}_{4}$Ga${}_{5}$ (brown points) compared to the spin-resolved total density of states (blue line) calculated for $U_{Ce1}=3$ eV and $U_{Ce2,Ce3}=2.25$ eV. (**b**) Total and partial DOS in Ce${}_{9}$Ru${}_{4}$Ga${}_{5}$, calculated as in panel (**a**), compared to the experimental XPS data.

The electronic bands in Ce${}_{9}$Ru${}_{4}$Ga${}_{5}$, calculated assuming the correlation energy U=1.5 eV and 2.25 eV, are shown in Figure 3 in a form of the total density of states (TDOS). In addition, the calculations were performed for a model in which different *U* values were attributed to distinct Ce atoms, and Figure 3 displays the result obtained setting U=3 eV for the Ce1 atom and U=2.25 eV for the Ce2 and Ce3 atoms. As can be inferred from the figure, the DFT data hardly depend on *U*, except a narrow range of binding energies −1 eV $<E<E_{F}$. Figure 4a shows the spin-resolved TDOS calculated for the latter values of *U* compared with the valence band of of Ce${}_{9}$Ru${}_{4}$Ga${}_{5}$ determined experimentally. Figure 4b, with an expanded energy scale, presents the same theoretical and XPS data together with the partial TDOS due to the particular atoms in the unit cell. Clearly, all the features present in the XPS spectrum are properly reproduced in the computed data. The main contribution due to the Ru 4d states is distributed between $E_{F}$ and the binding energy of 4 eV. In turn, the Ga 4*p* states form bands located near the binding energy of about 6 eV. The Ce 4f states are responsible for a broad and fairly weak feature near $E_{F}$. At the binding energy of about 17 eV and 19 eV, the calculated Ce 5p electronic states show SO-separated features, which are displaced in respect to the measured data by ~1 eV. The discrepancy can be attributed to Ce 5d-electron correlations, which usually shift the calculated Ce 5p states to lower binding energies [26,37,38].

The main numerical results of the performed PBEsol+U calculations are listed in Table 3. For various *U*, the mean occupancy of the 4f shell of the Ce1 atoms is close to 1, while $n_{f}$ computed for both the Ce2 and Ce3 atoms is notably smaller than 1. The effect of *U* on the obtained 4f electron count is almost negligible. Taking into account the multiplicity of the particular Ce sites, one obtains an average filling of the 4f shell in Ce${}_{9}$Ru${}_{4}$Ga${}_{5}$ equal to 0.86–0.89, in perfect agreement with the experimental result $n_{f}\approx 0.88$ (see above). The calculated total magnetic moment $m_{Ce1}$ amounts to about 1 $\mu_{B}$, regardless of the value of *U*, whereas the magnetic moment found at the Ce2 and Ce3 sites is significantly smaller, namely, $m_{Ce2}\approx 0.3$ $\mu_{B}$ and $m_{Ce3}\approx 0.5$ $\mu_{B}$.

**Figure 5:**
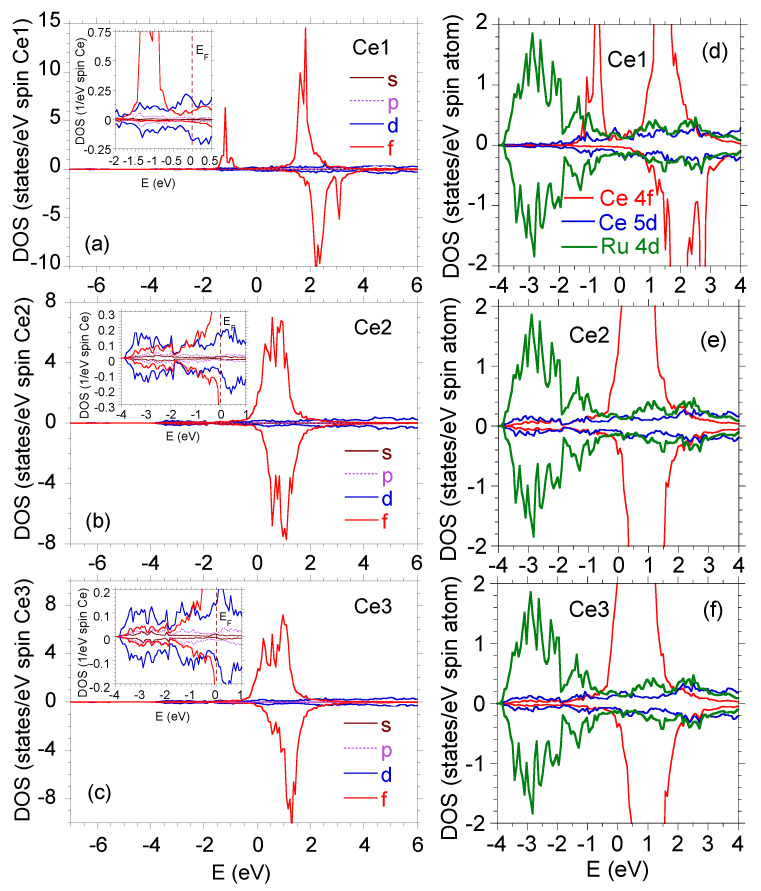
Spin-resolved density of states in Ce${}_{9}$Ru${}_{4}$Ga${}_{5}$ due to (**a**) Ce1,(**b**) Ce2, and (**c**) Ce3 atoms. The density of states (DOS) calculations were performed for the correlation parameters *U* = 3 eV for Ce1, and *U* = 2.25 eV for Ce1 and Ce2. The insets present in details the 5d contributions. The right panels (**d**–**f**) show partial density of states in Ce${}_{9}$Ru${}_{4}$Ga${}_{5}$ due to Ce 5d and Ru 4d electrons at Ce1 (**d**), Ce2 (**e**), and Ce3 (**f**) sites. The DOS calculations were performed for the correlation parameters *U* = 3 eV for Ce1 atom, and *U* = 2.25 eV for Ce2 and Ce3 atoms.

The fractional valence of the Ce2 and Ce3 ions likely results from the on-site hybridization effect as well as some intersite hybridization between the Ce 4f and Ru *d*-electron states. As can be inferred from Figure 5a–c, the on-site f–c hybridization causes a significant increase in the number of Ce2 and Ce3 5d-electron states, whereas the Ce1 4f electrons remain well localized at the binding energy of about 1 eV. At the same time, the DFT calculations clearly revealed strong inter-band hybridization of the Ce 5d and Ru 4d electron states for the Ce2 and Ce3 atoms, whereas the latter effect is negligibly small for the Ce1 atom (see Figure 5d–f).

**Figure 6:**
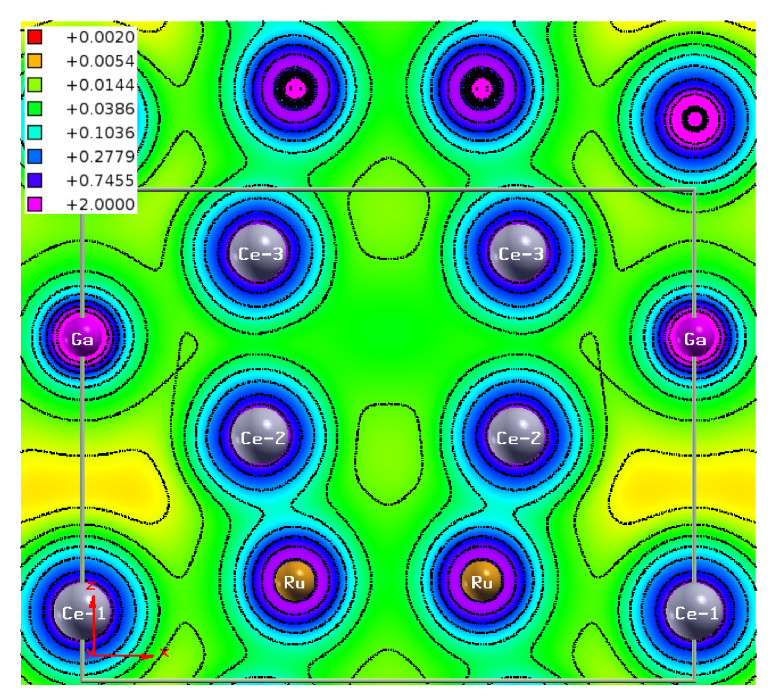
Electron densities ρ(r)/e (in (au)${}^{-3}$) visualized for the crystallographic plane (010) of the crystal structure of Ce${}_{9}$Ru${}_{4}$Ga${}_{5}$. The projection of the unit cell is outlined by gray lines.

In order to visualize the intersite hybridization and the atomic bonds in the unit cell of Ce${}_{9}$Ru${}_{4}$Ga${}_{5}$, we calculated the charge densities (setting $U_{Ce1}=3$ eV and $U_{Ce2,Ce3}=2.25$ eV). Figure 6 displays the electron density map within the crystallographic (010) plane. The map clearly shows almost isotropic distribution of valence electrons around the Ce1 and Ga atoms. In contrast, the charge distribution near the Ce2, Ce3, and Ru atoms is strongly anisotropic with strong accumulation of the electronic density along the bonds Ce2-Ru and Ce3-Ru. The strongest covalent bonding occurs between the Ce2 and Ru atoms, in concert with the crystal structure refinement, which revealed abnormally short Ce2-Ru interatomic distance [16]. Thus, the DFT calculations fully corroborated the scenario developed before [16,17], in which the IV behavior in Ce${}_{9}$Ru${}_{4}$Ga${}_{5}$, evidenced in the spectroscopic and thermodynamic properties of the compound, can be associated primarily with the Ce2 atoms.

## 5. Conclusions

The XPS experiment performed for Ce${}_{9}$Ru${}_{4}$Ga${}_{5}$ confirmed the fractional valence of the Ce ions, noticed before in the $L_{3}$ XANES spectroscopy [16] and bulk thermodynamic measurements [17]. The compound forms with a crystallographic unit cell that hosts three inequivalent Wyckoff positions for Ce atoms, thus the experimentally derived filling of the 4f shell ($n_{f}\approx 0.88$) was an average over those three sites. The DFT calculations allowed for inspecting the 4f electron counts at each Ce atom. The results indicated that the Ce1 ion located at the 2a site is trivalent ($n_{f}$ is close to 1). In contrast, the Ce2 and Ce3 ions, placed at the 8d sites, were found intermediate valent with $n_{f}$ notably smaller than 1. The ab initio calculated mean occupation of the 4f shell in Ce${}_{9}$Ru${}_{4}$Ga${}_{5}$ is 0.86–0.89, which is in very good agreement with the experimental finding.

The electronic instability of the 4f shell in the Ce2 ion gives rise to the IV character of the compound, established before in the study on its low-temperature bulk physical properties [17]. Most remarkably, the IV features were found to coexist with a long-range antiferromagnetic (AFM) ordering that sets in below $T_{N}$ = 3.7 K [17]. As suggested by our group in an earlier study [17] these two phenomena are spatially separated, i.e., they develop in different Ce ions sublattices. The present DFT results have corroborated such a scenario. Due to dissimilar strength of the intra-site band hybridization, the calculated magnetic moments are distinctly different for Ce1 (∼1 $\mu_{B}$/atom), Ce2 (∼0.3 $\mu_{B}$/atom), and Ce3 (∼0.5 $\mu_{B}$/atom). Therefore, it is reasonable to attribute the AFM state principally to the Ce1 ions, with a possible contribution due to the Ce3 ions, while the Ce2 ions remain nonmagnetic. The energy $\Delta_{fc}∼200$ meV is for Ce${}_{9}$Ru${}_{4}$Ga${}_{5}$ quite large, however, $V_{fc}={\left(\frac{\Delta_{fc}}{\pi N(E_{F})}\right)}^{1/2}∼44$ meV. One can also estimate the coupling constant $J_{fc}=\frac{2V_{fc}^{2}}{|E_{f}-E_{F}|}\approx 5$ meV [1] between the nearest Ce magnetic moments, which well correlates with low $T_{N}$ temperature-scale. In order to verify this tempting conjecture, neutron diffraction experiment is compulsory. Undoubtedly, the coexistence of intermediate valent and trivalent cerium ions makes Ce${}_{9}$Ru${}_{4}$Ga${}_{5}$ an interesting material for further comprehensive experimental and theoretical investigations.

## Figures and Tables

**Table 1:** Relaxed atomic positions in Ce${}_{9}$Ru${}_{4}$Ga${}_{5}$. Calculations were performed assuming the experimental lattice parameters a=b=10.1132 Å and c=8.1212 Å.

Wyckoff	Atom		Coordinates	
Position		x	y	z
2a	Ce1	0.000000	0.000000	0.141812
8d	Ce2	0.290732	0.000000	0.498706
8d	Ce3	0.287464	0.000000	0.871542
8d	Ru	0.347568	0.000000	0.203546
2a	Ga1	0.000000	0.000000	0.699153
8c	Ga2	0.217667	0.217667	0.178952

**Table 2:** Interatomic distances (Å) in the crystal structure of Ce${}_{9}$Ru${}_{4}$Ga${}_{5}$.

Ce1	Atom	Distance	Ce2	Atom	Distance	Ce3	Atom	Distance
	4Ga2	3.1355		Ru	2.4237		3Ru	2.8233
	Ga1	3.3769		Ce3	3.0114		Ru	2.9531
	4Ce3	3.4661		2Ru	3.0792		Ce2	3.0114
	4Ru	3.6321		2Ce3	3.1721		2Ce2	3.1721
	4Ce2	4.3802		2Ga2	3.2187		Ga1	3.2437
	Ga1	4.7443		Ga1	3.3633		2Ga2	3.2791
				2Ga2	3.5047		2Ga2	3.3838
				Ce2	4.1355		Ce1	3.4661
				2Ce2	4.2269		2Ce3	4.0909
				Ru	4.2978		Ce3	4.3277
				Ce1	4.3802		Ru	4.5993

**Table 3:** Results of the LSDA+U calculations performed for Ce${}_{9}$Ru${}_{4}$Ga${}_{5}$ with different values of the correlation energy *U*, where $n_{f}$ stands for the number of 4f electrons and *m* is the total magnetic moment per atom.

*U* (eV)		$U_{\mathrm{Ce}1,\mathrm{Ce}2,\mathrm{Ce}3}$ = 1.5		$U_{\mathrm{Ce}1,\mathrm{Ce}2,\mathrm{Ce}3}$ = 2.25		$U_{\mathrm{Ce}1}$ = 3 $U_{\mathrm{Ce}2,\mathrm{Ce}3}$ = 2.25
$N(E_{F})$ (eV f.u.)${}^{-1}$		31.32		31.32		34.26
Atom	$n_{f}$	m ($\mu_{B}$)	$n_{f}$	m ($\mu_{B}$)	$n_{f}$	m ($\mu_{B}$)
Ce1	0.9833	0.9644	0.9880	0.9951	0.9873	1.0149
Ce2	0.8860	0.2589	0.8446	0.2808	0.8451	0.3099
Ce3	0.8732	0.4031	0.8309	0.4602	0.8423	0.5295
Ru		−0.0396		−0.0446		−0.0477
Ga1		0.0014		0.0023		0.0027
Ga2		0.0013		0.0024		0.0035
